# Detection of leptomeningeal angiomas in brain MRI of Sturge-Weber syndrome using multi-scale multi-scan Mamba

**DOI:** 10.3389/fnins.2025.1699700

**Published:** 2025-11-18

**Authors:** Weiqun Bao, Chenghao Xue, Ruisheng Su, Xindan Hu, Yuanning Li, Xiaoqiang Wang, Tao Tan, Dake He, Lin Xu

**Affiliations:** 1 Department of Pediatric Neurology, Xinhua Hospital Affiliated to Shanghai Jiao Tong University School of Medicine, Shanghai, China; 2 School of Information Science and Technology, ShanghaiTech University, Shanghai, China; 3 Department of Biomedical Engineering, Eindhoven University of Technology, Eindhoven, Netherlands; 4 School of Biomedical Engineering, ShanghaiTech University, Shanghai, China; 5 Department of Pediatric Neurosurgery, Xinhua Hospital Affiliated to Shanghai Jiao Tong University School of Medicine, Shanghai, China; 6 Faculty of Applied Sciences, Macao Polytechnic University, Macao, Macao SAR, China

**Keywords:** Sturge-Weber syndrome, leptomeningeal angiomas, magnetic resonance imaging, Mamba, multi-scale multi-scan

## Abstract

**Objectives:**

Sturge-Weber syndrome (SWS) is a congenital neurological disorder occurring in the early childhood. Timely diagnosis of SWS is essential for proper medical intervention that prevents the development of various neurological issues. Leptomeningeal angiomas (LA) are the clinical manifestation of SWS. Detection of LA is currently performed by manual inspection of the magnetic resonance images (MRI) by experienced neurologist, which is time-consuming and lack of inter-rater consistency. The aim of the present study is to investigate automated LA detection in MRI of SWS patients.

**Methods:**

A Mamba-based encoder-decoder architecture was employed in the present study. Particularly, a multi-scale multi-scan strategy was proposed to convert 3-D volume into 1-D sequence, enabling capturing long-range dependency with reduced computation complexity. Our dataset consists of 40 SWS patients with T1-enhanced MRI. The proposed model was first pre-trained on a public brain tumor segmentation (BraTS) dataset and then fine-tuned and tested on the SWS dataset using 5-fold cross validation.

**Results and conclusion:**

Our results show excellent performance of the proposed method, e.g., Dice score of 91.53% and 78.67% for BraTS and SWS, respectively, outperforming several state-of-the-art methods as well as two neurologists. Mamba-based deep learning method can automatically identify LA in MRI images, enabling automated SWS diagnosis in clinical settings.

## 1 Introduction

Sturge-Weber syndrome (SWS) is a rare congenital neurological disorder with an incidence rate of approximately 1 in 20,000 to 50,000 live births (Comi, 2007). It is often associated with facial port-wine stains (PWS), glaucoma, and ipsilateral leptomeningeal angiomas (LA) (Sudarsanam and Ardern-Holmes, 2014; Thomas-Sohl et al., 2004). SWS can lead to various neurological issues such as seizures, hemiparesis, headaches, and cognitive impairments, particularly in children under five years (Sujansky and Conradi, 1995). Given the high susceptibility to seizures in this age group, early detection of SWS and proper medical intervention are crucial. Although facial PWS is a major characteristic of SWS, not everyone with facial PWS suffers from SWS (Thomas-Sohl et al., 2004). Therefore, it is recommended that children with PWS undergo imaging evaluation, such as magnetic resonance imaging (MRI), to determine whether they have SWS (Bar et al., 2020).

Clinical manifestation of SWS is the presence of intracranial vascular anomaly, i.e., LA (Ramirez and Jülich, 2024). Currently, the identification of LA relies on visual inspection of the imaging data, particularly MRI, by experienced clinicians. However, the expertise of experienced doctors may not always be readily available, and manual labeling of lesions is time-consuming. Therefore, an automatic diagnosis tool is required for accurate and efficient diagnosis of SWS.

With the advance in deep learning, encoder-decoder-based frameworks such as U-Net and its variants have become the dominant architectures for medical image segmentation (Ronneberger et al., 2015; Çiçek et al., 2016; Soh and Rajapakse, 2023; Chen et al., 2025), playing a key role in computer-aided diagnosis (CAD) systems. U-Net primarily utilizes the encoder-decoder structure with convolutional neural networks (CNNs) in each layer to extract hierarchical image features, enabling precise segmentation of the image in pixel level (Çiçek et al., 2016). Moreover, nnU-Net automates hyperparameter tuning and data preprocessing for U-Net, and therefore enhances the performance in many segmentation tasks (Isensee et al., 2021). However, while CNNs are efficient in feature extraction, they are constrained by their local receptive fields, making it difficult to capture long-range dependencies that are crucial for tasks where global context is essential (Soh and Rajapakse, 2023).

In addition to CNNs, Transformer has emerged as a powerful alternative, demonstrating exceptional ability to model global relationships and capture long-range dependencies (Dosovitskiy et al., 2021; Raghu et al., 2021). Vision Transformer(ViT) employs the transformer architecture for image processing, capturing global dependencies in images through self-attention mechanisms. Nevertheless, their quadratic complexity associated with the length of the input sequence renders them computationally intensive and impractical for handling high-dimensional medical images (Gu and Dao, 2024). This computational burden hinders their applicability in real-world settings with limited resources.

Mamba has been recently proposed to capture long-range dependence with reduced computational burden. It is built on the state space models (SSMs) with optimized state space matrices (Gu and Dao, 2024), and has shown promising performance in natural language processing and also been adapted for computer vision tasks (Zhu et al., 2025). However, as the SSMs work initially on 1-D sequence, adapting Mamba for image tasks requires to convert the 2- or 3-D images into 1-D sequence. Several strategies have been proposed for such adaptation. U-Mamba flattens image patches using a original scan order, i.e., row by row sequentially (Ma et al., 2024). VMamba introduces a cross-scan module to decompose 2-D images into four distinct 1-D scanning paths (horizontal, vertical, and their reverse directions) to better preserve spatial relationships (Liu Y. et al., 2024). Swin-UMamba integrates a shifted window partitioning strategy (inspired by Swin Transformers) with VMamba's directional scanning, while also leveraging ImageNet pre-training for enhanced feature representation (Liu et al., J. 2024). In addition, SegMamba proposes a tri-orientated scanning scheme for 3-D images, i.e., scanning along axial, sagittal, and coronal planes separately to capture volumetric dependencies (Xing et al., 2024).

Despite their wide application, deep-learning-based CAD techniques have not yet been applied to the diagnosis of SWS. The aim of the present study is therefore to develop a deep-learning-based tool for automatic detection of LA in the brain MRI of SWS patients. The study is designed as a retrospective investigation of a clinical dataset consisting of 40 SWS patients. The encoder-decoder structure and Mamba model are considered. In order to deal with the random location of LAs in MRI scans, a novel multi-scale multi-scan (MSMS) strategy is proposed to convert 3-D volumes into 1-D sequences. Besides, due to the rareness of the disease and thus limited sample size, transfer learning is considered by pre-training the model with a public dataset, i.e, the brain tumor segmentation (BraTS) benchmark dataset, and then fun-tuning and testing it on our SWS dataset. The performance of the proposed method is compared with CNNs, Transformers, and state-of-the-art (SOTA) Mamba-based models in terms of a number of evaluation metrics.

The main novelty and contributions of the present study are summarized hereafter.

- An encoder-decoder deep learning method was employed, for the first time, for automatic identification of LA in the brain MRI images, and thus for automated diagnosis of SWS.
- Mamba model is embedded in the encoder in order to capture long-rang dependency in 3-D volumes.
- A multi-scale multi-scan strategy is proposed in the Mamba model to extract multi-scale futures along different directions of the 3-D volume.
- Pre-training on the BraTS dataset is employed to address the challenge of limited sample size.

## 2 Material and methods

### 2.1 Dataset and pre-processing

This study was designed as a retrospective study of existing data collected during clinical practice at Xinhua Hospital Affiliated to Shanghai Jiao Tong University School of Medicine (XH-SJTU-SM). Patients diagnosed as SWS between June 2008 and August 2024 were considered. The exclusion criteria include: (a) younger than 3 months; (b) syndrome type II, i.e., without LA; (c) missing clinical information or poor-quality images impeding manual annotation. Consequently, 40 SWS patients (13 girls and 27 boys, age between 5-696 months) were involved in this study. The study protocol was approved by the Ethics Committee at XH-SJTU-SM with the number XHEC-D-2024-090. Written informed consent of each subject was waived by the ethics committee due to its retrospective nature.

**Figure 1:**
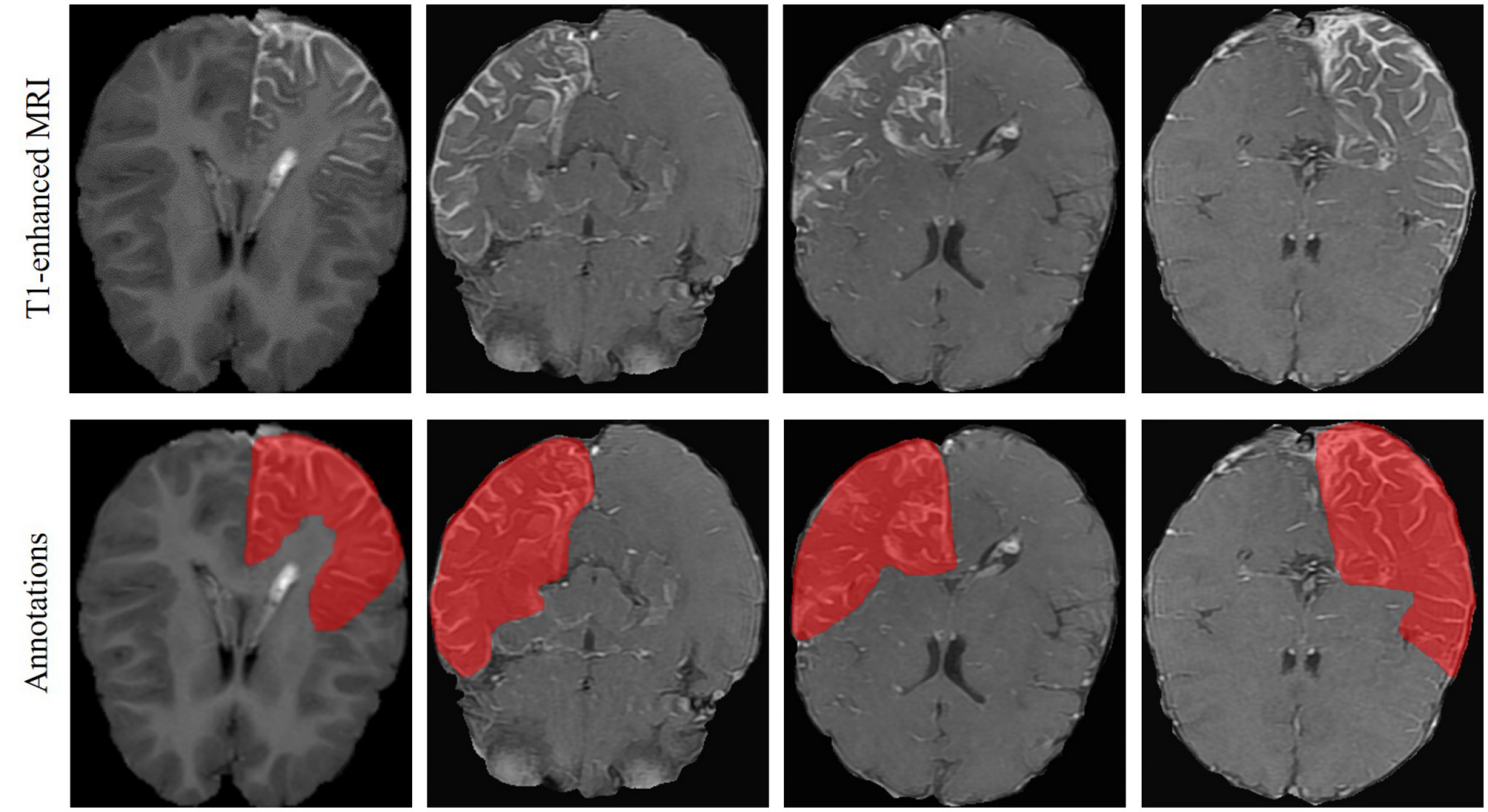
LA in T1-enhanced brain MRI of the SWS patients and the corresponding annotations.

T1-weighted MRI images were analyzed in the present study. They were recorded using different scanners, i.e., Siemens, Philips, and General Electric (GE), with two different magnetic field strengths, i.e., 1.5T and 3T. Details of the scanners and their parameters are summarized in Table 1. In each MRI image, the brain areas containing LA were annotated as the ground truth by one experienced neuroradiologist from XH-SJTU-SM using the well-known ITK-SNAP toolbox (Yushkevich et al., 2006). An representative examples of LA in T1-enhanced MRI and the corresponding annotations is shown in Figure 1. Note that marking only the abnormal intracranial vessels in the MRI image was quite difficult and therefore was not considered in the present study.

**Table 1:** MRI scanners and corresponding parameters.

**Scanner**	**NOP**	**TR/TE**	**FOV**	**MZ**	**ST**
Si 1.5T	3	1,600/8.9	200 × 200	256 × 180	5
Ph 3T	5	1,800/20	230 × 187	260 × 189	5
GE 3T	17	1,750/24	200 × 200	320 × 224	5
GE 1.5T	15	520/9.9	240 × 240	320 × 160	5

Si, Siemens; Ph, Philips; NOP, number of patients; TR, repetition time (ms); TE, echo time (ms); FOV, Filed of view (mm^2^); MZ, Matrix size (pixel^2^); ST, Slice thickness (mm).

In each MRI image, the skull was first removed using the FSL toolbox (Yushkevich et al., 2006) to avoid its affects on the identification of contrast-enhanced LA. Then the images recorded with different scanners and protocols were resampled to uniform physical spacing of 0.47 mm × 0.47 mm × 6.5 mm. In addition, each image was cropped to contain only the brain area. Due to different brain sizes of the subjects, the largest size of the cropped image was determined as 320 pixels × 320 pixels, and all cropped images were zero-padded to this unique size to keep consistency for further analysis.

Furthermore, a harmonization technique (Tan et al., 2023) was applied to the cropped images to compensate the influence of different scanners and imaging parameters. To this end, a high-quality MRI scan was first selected among all the images as a reference image (*I*_ref_) based on visual inspection. Then each brain image *I* was smoothed using *k* Gaussian kernels with different standard deviations:


$$\begin{array}{l} I^{\left(k\right)}=G_{\sigma_{k}}*I, \end{array}$$ (1)

where *G*_σ_*k*__ denotes the *k*^*th*^ Gaussian kernel, and σ_*k*_ = 0, 2, 4, 6, for *k* = 0, 1, 2, 3, respectively. Note that σ_0_ = 0 corresponds to the original (non-smoothed) image.

Each smoothed image *I*^(*k*)^ was then normalized with respected to the reference image, given by


$${\tilde{I}}^{\left(k\right)}=\frac{I^{\left(k\right)}-\mu^{\left(k\right)}}{\sigma^{\left(k\right)}}\cdot \sigma_{\text{ref}}^{\left(k\right)}+\mu_{\text{ref}}^{\left(k\right)},$$ (2)

where μ and σ indicates respectively mean and standard deviation, and $\mu_{\text{ref}}^{\left(k\right)}$, $\sigma_{\text{ref}}^{\left(k\right)}$ are computed from the smoothed reference image $I_{\text{ref}}^{\left(k\right)}$ calculated as $I_{\text{ref}}^{\left(k\right)}=G_{\sigma_{k}}*I_{\text{ref}}$. Finally, a harmonized image was obtained as


$$\begin{array}{l} I_{\text{harm}}=\overset{3}{\underset{k=0}{\sum }}{\tilde{I}}^{\left(k\right)}. \end{array}$$ (3)

### 2.2 Mamba-based segmentation framework

#### 2.2.1 Overall framework

**Figure 2:**
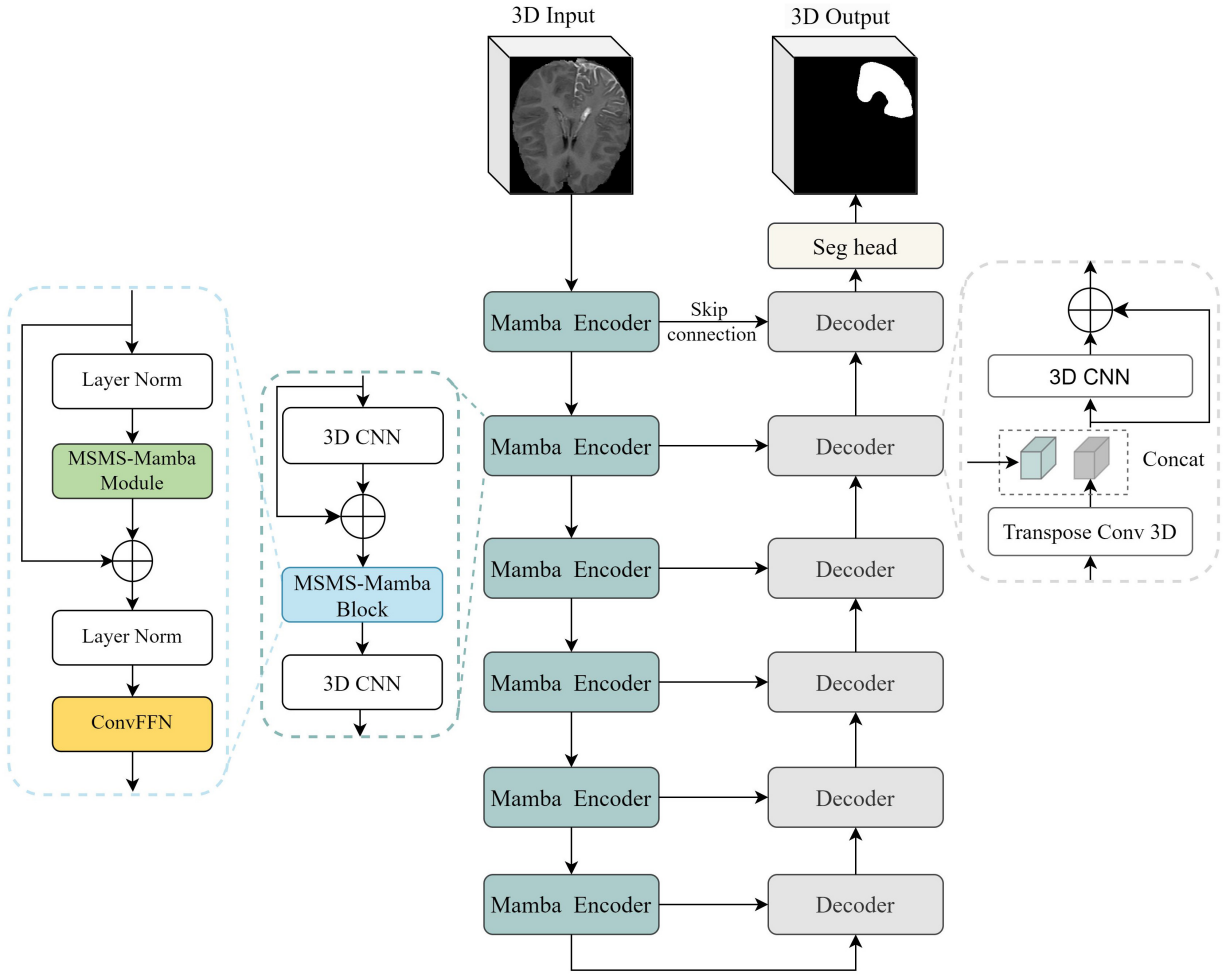
The overall architecture of the proposed segmentation framework.

The overall architecture of the proposed segmentation framework is illustrated in Figure 2. It is based on the U-Net structure with six encoding and decoding layers. In the encoder, each layer consists of two 3D CNN modules and a multi-scale multi-scan (MSMS) Mamba block in between. The first CNN is used to extract 3-D features from the input volume. It is composed of a depthwise convolution followed by a pointwise convolution for efficient channel-wise feature mixing (Zhang et al., 2019), followed by an instance normalization (Ulyanov et al., 2016), and a Leaky ReLU activation function (Xu et al., 2020). The MSMS-Mamba block is connected to the output of the residual CNN to enhance the model's ability to capture long-range dependencies and spatial correlations. The second 3D CNN is employed as an efficient alternative to traditional max-pooling to progressively down-sample the feature map in each encoding layer.

In the decoder, each layer comprises a 3D CNN module and an up-sampling module, i.e., transposed 3D convolution. The 3D CNN module is the same as the encoder. The up-sampling module leverages 3D transposed convolutions to progressively restore the spatial resolution of feature maps. At the end of the encoder, i.e., the final layer, a segmentation head with 1 7 1 convolution is employed to perform pixel-wise classification, producing the final segmentation output. Besides, following established practices in medical image segmentation (Isensee et al., 2021; Hatamizadeh et al., 2021), skip connections are introduced between corresponding encoding and decoding layers to retain spatial detail and low-level features.

**Figure 3:**
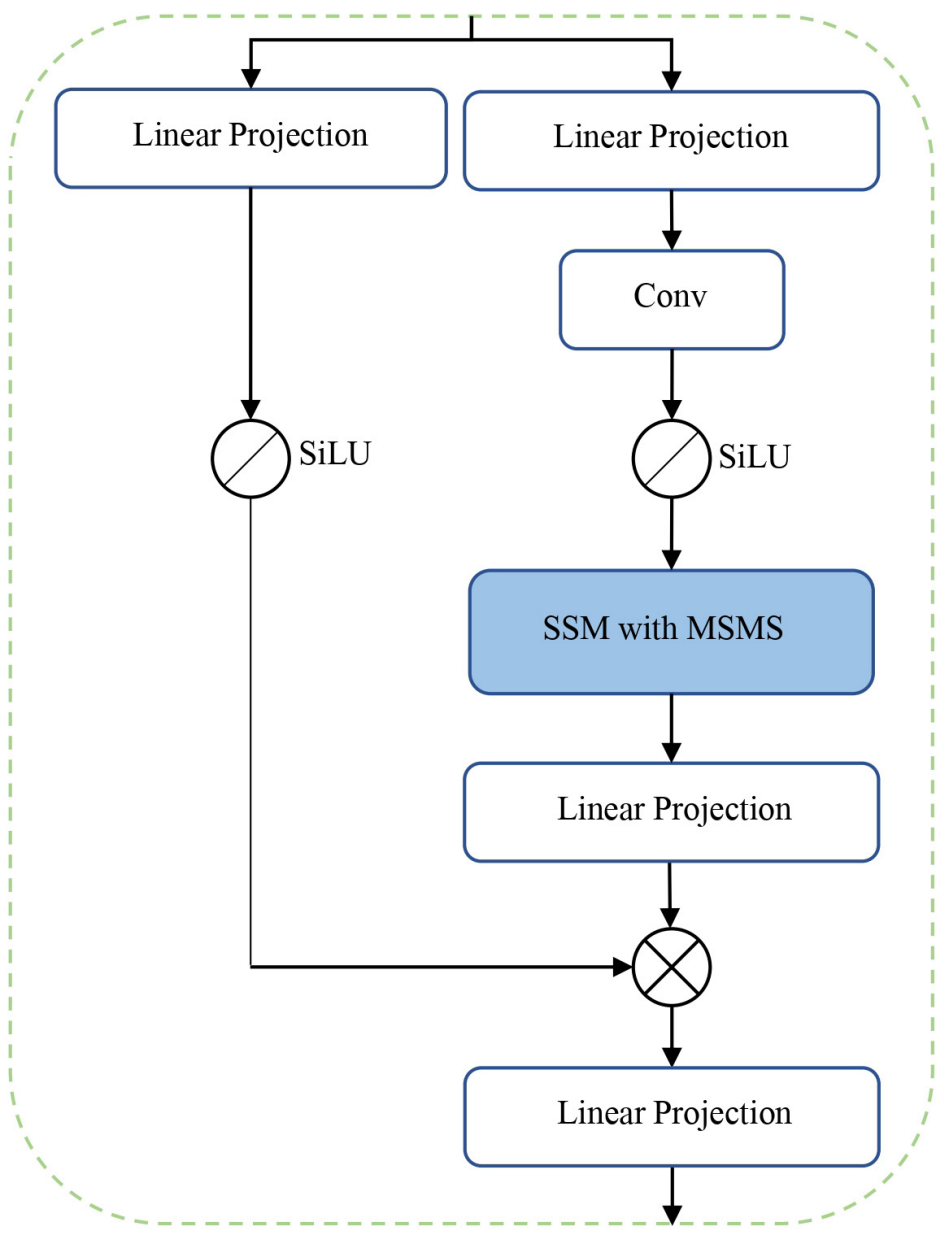
MSMS-Mamba module.

**Figure 4:**
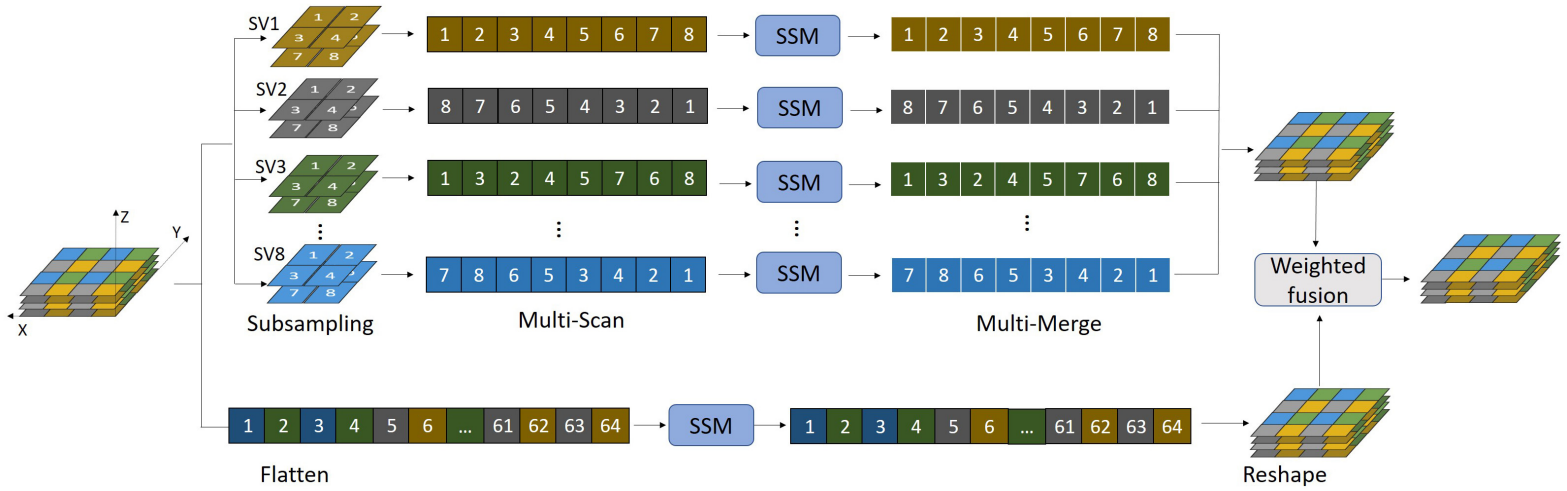
SSM with multi-scale multi-scan strategy.

The main novelty and contribution of the present work lies in the integration of the MSMS-Mamba block in the encoder to enhance long-range dependency modeling and improve feature fusion. The scheme of the MSMS-Mamba block is shown in the left of Figure 2. It is composed of four consecutive components: an initial layer normalization, the MSMS-Mamba module for long-range dependency and multi-scale modeling, a second layer normalization, and a convolutional feed-forward network (Shi et al., 2024) for feature refinement. Among the four components, the MSMS-Mamba module is the key of the MSMS-Mamba block, whose scheme is shown in Figure 3. It is built based on a SSM model with dedicated MSMS strategy, as shown in Figure 4. Details of the SSM model and the MSMS strategy are introduced hereafter.

#### 2.2.2 Selective state space model

SSM is a class of sequence-to-sequence architecture that has undergone significant evolution in recent years. The development of SSMs (Gu et al., 2022) originates from their foundational formulation as a linear time-invariant (LTI) system, which maps an input sequence *x*(*t*) ∈ ℝ^*L*^ to an output sequence *y*(*t*) ∈ ℝ^*L*^ through a hidden state *h*(*t*) ∈ ℂ^*N*^. The continuous form of SSMs is defined as:


$$\begin{array}{l} h^{\prime }\left(t\right)=Ah\left(t\right)+Bx\left(t\right),\\ \;y\left(t\right)=Ch\left(t\right), \end{array}$$ (4)

where ***A*** ∈ ℂ^*N*×*N*^, ***B*** ∈ ℂ^*N*^, and ***C*** ∈ ℂ^*N*^ are the system matrices, and *h*(*t*) ∈ ℝ^*N*^ represents the implicit latent state.

To make SSMs computationally tractable for discrete-time sequences, the bilinear transform method is employed to discretize the continuous ordinary differential equations. This results in the discrete form of SSMs,


$$\begin{array}{l} h\left[n\right]=\bar{A}h\left[n-1\right]+\bar{B}x\left[n\right],\\ y\left[n\right]=\bar{C}h\left[n\right], \end{array}$$ (5)

where $\bar{A}={\left(I-\Delta /2\cdot A\right)}^{-1}\left(I+\Delta /2\cdot A\right)$, $\bar{B}={\left(I-\Delta /2\cdot A\right)}^{-1}\Delta B$, and $\bar{C}=C$. Here, Δ denotes the step size for converting a continuous sequence into a discrete sequence (Gu et al., 2022).

The discrete SSM can be further expressed in a convolutional form, shown as


$$\begin{array}{l} y\left[n\right]=C{\bar{A}}^{n}\bar{B}x\left[0\right]+C{\bar{A}}^{n-1}\bar{B}x\left[1\right]+\ldots \\ \;+C\bar{A}\bar{B}x\left[n-1\right]+C\bar{B}x\left[n\right],\\ \;\bar{K}=\left(C\bar{B},C\bar{A}\bar{B},\ldots ,C{\bar{A}}^{n-1}\bar{B},C{\bar{A}}^{n}\bar{B}\right),\\ \;y=\bar{K}*x. \end{array}$$ (6)

The matrices $\bar{A}$, $\bar{B}$, and $\bar{C}$ remain fixed within every iteration. This allows pre-computing the convolution kernel $\bar{K}$, significantly speeding up the training stage, as reported in Gu et al. (2022).

SSMs are the core of a Mamba model. However, the pre-computing of $\bar{K}$ results in a static representation, where the same matrices are applied to all tokens regardless of the input content, limiting the model's ability to perform context-aware reasoning. To address this limitation, the Mamba model (Gu and Dao, 2024) introduces a selective mechanism to SSMs, making the system matrices input-dependent. This is achieved by constraining the system matrixes *B*, *C*, and Δ as linear projection of the input sequence *x* ∈ ℝ^*d*^ using a weight matrix *W* ∈ ℝ^*d*×*N*^. This makes *B*, *C*, and Δ input-dependent. Consequently, the input dependency of Δ causes $\bar{A}$ to also become input-dependent, rendering the pre-computed $\bar{K}$ inapplicable. To address this, a parallel scanning algorithm is proposed in Mamba for efficient computation of these matrices (Gu and Dao, 2024).

#### 2.2.3 Multi-scale multi-scan strategy

Mamba is initially designed for processing 1-D series, but can be adapted for image processing tasks by serializing the 2-D or 3-D image into a 1-D sequence through flattening operation. However, directly applying Mamba to flattened 3-D medical images leads to limited receptive fields, hindering the model's ability to effectively capture spatial correlations in higher-dimensional data. This limitation becomes particularly evident in 3-D medical imaging, where preserving spatial structure is essential for accurate analysis. To address this challenges, we propose a MSMS strategy to convert our 3-D MRI data into 1D sequence, which is designed as a dual-branch structure in order to extract multi-scale features, as shown in Figure 4. The upper branch performs feature extraction across different directions through a combination of sub-sampling and multi-scan operations. Meanwhile, the parallel lower branch maintains the original resolution to preserve fine-grained spatial information.

In the upper branch, sub-sampling is first employed to divide the input 3D volume *V* into several smaller sub-volumes (SVs) in order to alleviate long-range forgetting (Shi et al., 2024). To this end, the 3D volume *V* is uniformly partitioned into *N* segments along each axis, resulting in *N*^3^ basic elements: $V={\left\{x_{i};y_{j};z_{k}\right\}}_{i,j,k=1}^{N}$. In the present study, *N* = 4 thus 64 basic elements are obtained. Then 8 SVs can be derived, with each consisting of 8 basic elements sampled from the entire volume (two elements from each axis), as shown in Table 2. For instance, the SV_1_ in Table 2 is sampled from {*x*_1_, *x*_3_}, {*y*_1_, *y*_3_}, and {*z*_1_, *z*_3_}, denoted as


$$\begin{array}{l} \text{SV\_1}=V\left\{x_{1},x_{3};y_{1},y_{3};z_{1},z_{3}\right\}. \end{array}$$ (7)

Each SV is numbered in the same way as shown in Figure 4. Different scanning orders along different spatial directions are then employed to flatten each SV into 1-D sequence, as listed in Table 2. The multi-scan operation is designed to extract multi-scale features from each SV while effectively expanding the receptive field. Instead of bidirectional scanning, only unidirectional scanning for each SV is considered to achieve a trade-off between segmentation performance and computational complexity. However, different scanning orders are employed for different SVs, equalling to multi-directional scanning of the original volume with reduced spatial sample rate. Such diverse scanning patterns across different SVs enable complementary coverage among sequences, facilitating joint expansion of context without increasing computational cost. SSM is then applied to each flattened 1-D sequence, which is followed by the multi-merge operation, an inverse operation of multi-scan, reconstructs the 3-D volume based on the output of SSMs.

**Table 2:** Sub-volumes obtained by down-sampling and the corresponding scan order.

**SV**	**Sub-sample**	**Scan order**
SV_1	*V*{*x*_1_, *x*_3_; *y*_1_, *y*_3_; *z*_1_, *z*_3_}	[1, 2, 3, 4, 5, 6, 7, 8]
SV_2	*V*{*x*_2_, *x*_4_; *y*_1_, *y*_3_; *z*_1_, *z*_3_}	[8, 7, 6, 5, 4, 3, 2, 1]
SV_3	*V*{*x*_1_, *x*_3_; *y*_2_, *y*_4_; *z*_1_, *z*_3_}	[1, 3, 2, 4, 5, 7, 6, 8]
SV_4	*V*{*x*_2_, *x*_4_; *y*_2_, *y*_4_; *z*_1_, *z*_3_}	[8, 6, 7, 5, 4, 2, 3, 1]
SV_5	*V*{*x*_1_, *x*_3_; *y*_1_, *y*_3_; *z*_2_, *z*_4_}	[1, 5, 2, 6, 3, 7, 4, 8]
SV_6	*V*{*x*_2_, *x*_4_; *y*_1_, *y*_3_; *z*_2_, *z*_4_}	[8, 4, 7, 3, 6, 2, 5, 1]
SV_7	*V*{*x*_1_, *x*_3_; *y*_2_, *y*_4_; *z*_2_, *z*_4_}	[1, 2, 4, 3, 5, 6, 8, 7]
SV_8	*V*{*x*_2_, *x*_4_; *y*_2_, *y*_4_; *z*_2_, *z*_4_}	[7, 8, 6, 5, 3, 4, 2, 1]

### 2.3 Evaluation

#### 2.3.1 Evaluation on public BraTS dataset

The performance of the proposed segmentation framework was first evaluated on a public dataset from the BraTS2023 Challenge (Kazerooni et al., 2024), which includes 1251 3D brain MRI images with gliomas. This dataset was acquired from multiple institutions under standard clinical conditions using four modalities (T1, T1Gd, T2, and T2-FLAIR). Whole tumor (WT), tumor core (TC), and enhancing tumor (ET) were manually annotated by experienced medical experts. Only T1-enhanced images were considered, the same as our SWS dataset. We adopted a 7:1:2 split for training, validation, and testing, and trained the model for 1,000 epochs to ensure efficient learning and reliable performance evaluation.

#### 2.3.2 Evaluation on SWS dataset with pre-training strategy

While evaluating the model on the SWS dataset, a pre-training strategy was considered due to the limited subject size. In this scenario, the entire BraTS2023 dataset (Kazerooni et al., 2024) was adopted to pre-train the model for 1,000 epochs. Although gliomas in BraTS differ morphologically from SWS lesions, both share common segmentation principles—particularly the enhanced intensity patterns in T1-weighted images. Thus, pre-training on BraTS enables the model to leverage relevant priors and helps alleviate the limitations imposed by the small scale of the SWS dataset.

Given the fact that the spatial sampling distance of the SWS dataset in the z-axis is significantly larger than that of the x- and y-axis, down-sampling in the z-axis of the BraTS dataset was performed as it is originally equally sampled in all directions. After pre-training, the obtained model was fine-tuned and tested on the SWS dataset for 250 epochs, which is smaller than that of the BraTS dataset (1,000) due to the much smaller size of the SWS dataset. We utilized a 5-fold cross-validation strategy to evaluate the performance of the proposed method.

#### 2.3.3 Performance metrics

To quantitatively evaluate the segmentation performance of the proposed model on both datasets, the widely adopted dice similarity (DS) coefficient was employed as the primary metric, which measures the spatial overlap between model predictions and ground truth annotations. This metric has become the de facto standard in medical image segmentation due to its robustness in assessing volumetric agreement. However, note that in the present study the SWS dataset are severely imbalanced, i.e., much smaller LA regions as compared the non-LA regions. Such imbalance may lead to a low Dice score.

Therefore, other metrics such as sensitivity (Sen), specificity (Spe), balanced accuracy (BAcc), accuracy (Acc), Jaccard Index (JI) (Ronneberger et al., 2015), Kappa coefficient (Chmura Kraemer et al., 2002), volumetric similarity (VS), Matthews correlation coefficient (MCC) (Chicco and Jurman, 2020), and 95% Hausdorff distance (HD95) (Huttenlocher et al., 1993) were also calculated to measure the similarity between the segmented tumor and the ground truth in order to achieve a comprehensive understanding of the model performance.

#### 2.3.4 Implementation details

The PyTorch deep learning framework was utilized in our implementation. The training and evaluation processes were executed on a high-performance computing node featuring NVIDIA GeForce RTX 3090 graphics processor with CUDA 11.8 acceleration. We have implemented MSMSMamba on top of the well-established nnU-Net framework (Isensee et al., 2021). Its self-configuring feature has allowed us to focus on network design rather than other trivial details. The loss function was a combination of Dice loss and cross-entropy loss, defined as


$$\begin{array}{l} L_{\text{total}}=\frac{1}{2}\cdot L_{\text{CE}}+\frac{1}{2}\cdot L_{\text{Dice}}. \end{array}$$ (8)

The Cross Entropy Loss ℒ_CE_ and Dice Loss ℒ_Dice_ are respectively defined as


$$\begin{array}{l} L_{\text{CE}}=-\frac{1}{N}\overset{N}{\underset{i=1}{\sum }}\left[y_{i}\log\left(p_{i}\right)+\left(1-y_{i}\right)\log\left(1-p_{i}\right)\right], \end{array}$$ (9)

and


$$\begin{array}{l} L_{\text{Dice}}=1-\frac{2\overset{N}{\underset{i=1}{\sum }}p_{i}y_{i}+\epsilon }{\overset{N}{\underset{i=1}{\sum }}p_{i}+\overset{N}{\underset{i=1}{\sum }}y_{i}+\epsilon }, \end{array}$$ (10)

where *N* is the number of samples (or pixels), *y*_*i*_ ∈ {0, 1} denotes the ground truth label of the *i*-th sample, *p*_*i*_ ∈ [0, 1] denotes the predicted probability for the positive class, and ϵ is a small constant (e.g., 10^−6^) to prevent division by zero. The combined loss was supervised at each layer of the decoder to achieve deep supervision, as suggested in previous studies (Lee et al., 2015), with exponentially decayed weights (1/2^*i*^) assigned to lower-resolution outputs. The AdamW optimizer with a weight decay of 0.05 was considered (Liu Y. et al., 2024). A cosine learning rate decay was adopted with an initial learning rate of 0.001.

Detailed model parameters of the CNN and Mamba blocks are reported in Table 3. Note that each CNN block is composed of a depthwise convolution and a pointwise convolution, and only the parameters for depthwise convolution are shown here. The pointwise convolution employed a fixed kernel (1,1,1) with stride (1,1,1) in all layers. For the MSMS-Mamba blocks, the key parameters, i.e., state dimension, convolutional kernel size, and expansion ratio were set as default configuration in Mamba (Gu and Dao, 2024).

**Table 3:** Detailed model parameters of the proposed architecture.

**Layer**	**Encoder**						**Decoder**		
	**3D CNN** ^*^			**Mamba**			**Transposed Conv**		
	**Kernel**	**Stride**	**Channel**	*d*_state_	*d*_conv_	**ER**	**Kernel**	**Stride**	**Channel**
1	(3,3,1)	(1,1,1)	2 → 32	16	4	2	(2,2,1)	(1,1,1)	64 → 32
2	(3,3,1)	(2,2,1)	32 → 64	16	4	2	(2,2,1)	(1,1,1)	128 → 64
3	(3,3,1)	(2,2,1)	64 → 128	16	4	2	(2,2,1)	(1,1,1)	256 → 128
4	(3,3,3)	(2,2,2)	128 → 256	16	4	2	(2,2,2)	(2,2,2)	320 → 256
5	(3,3,3)	(2,2,2)	256 → 320	16	4	2	(2,2,2)	(2,2,2)	320 → 320
6	(3,3,3)	(2,2,1)	320 → 320	16	4	2	(2,2,1)	(2,2,1)	320 → 320

^*^Each CNN block is composed of a depthwise convolution and a pointwise convolution, and only depthwise convolution parameters are shown here. The pointwise convolution uses a fixed kernel (1,1,1) with stride (1,1,1) in all layers. *d*_state_: state dimension; *d*_conv_: convolution kernel size; ER, Expansion ratio.

## 3 Results

### 3.1 Segmentation results on BraTS dataset

**Figure 5:**
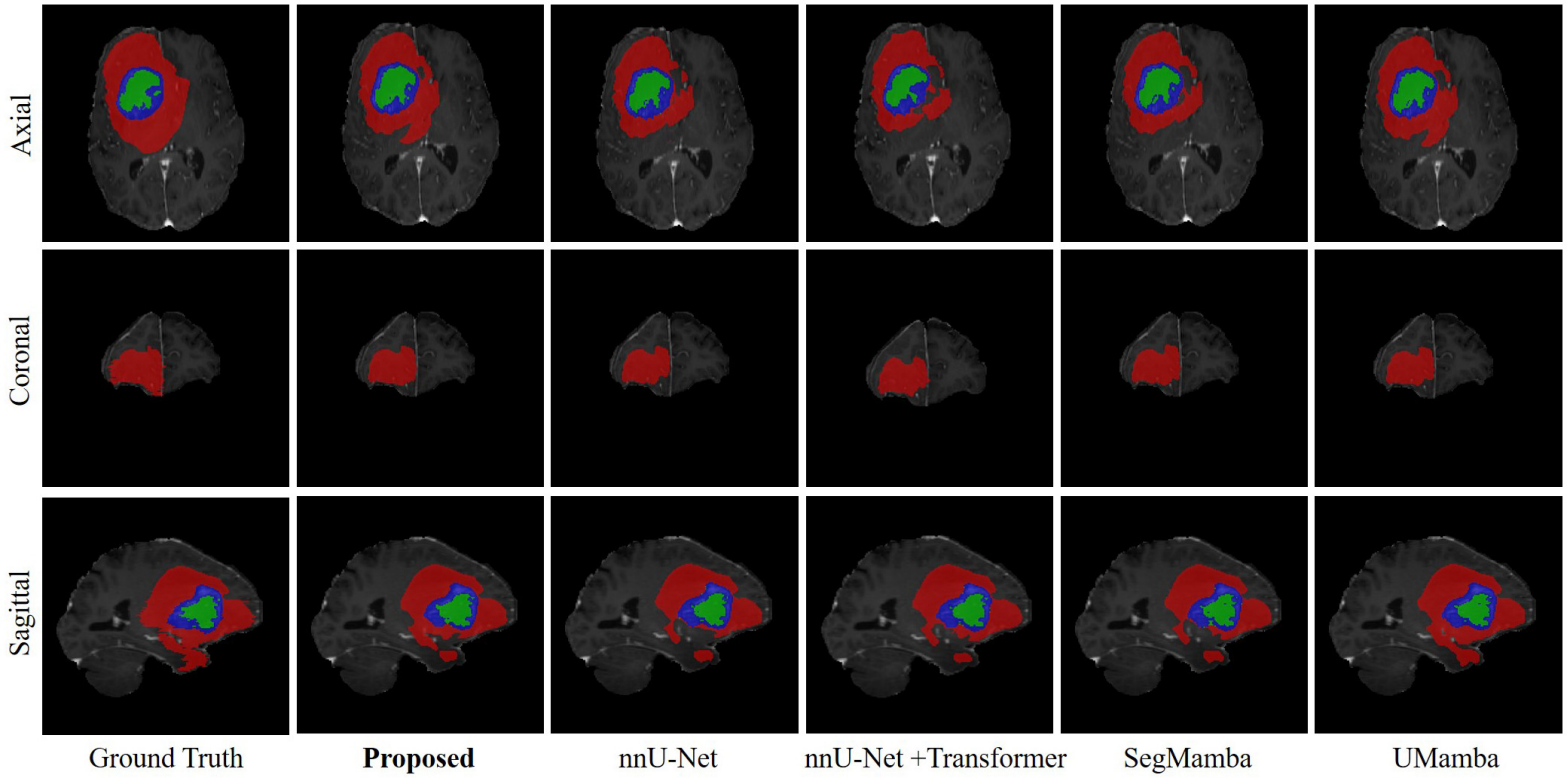
Example of the segmentation results on the BraTS dataset. Red, WT; Blue, TC; Green, ET.

Figure 5 shows an example of the segmentation results of the WT, TC, and ET on the BraTS dataset, produced by our model as well as several SOTA methods such as nnU-Net (Isensee et al., 2021), UMamba (Ma et al., 2024), and SegMamba (Xing et al., 2024), which are re-implemented in the present study based on the open-source code. All methods seem to produce very good segmentation results. However, the quantitative metrics over the entire testing set, showing in Table 4, suggest that our model outperforming nnU-Net, UMamba, and SegMamba except for Dice in TC and HD95 in ET.

**Table 4:** Segmentation results on the BraTS2023 dataset.

**Methods**	**WT**		**TC**		**ET**		**Average**	
	**Dice (%)**	**HD95 (mm)**	**Dice (%)**	**HD95 (mm)**	**Dice (%)**	**HD95 (mm)**	**Dice (%)**	**HD95 (mm)**
SegresNet^†^	92.02	4.07	89.10	4.08	83.66	3.88	88.26	4.01
UX-Net^†^	93.13	4.56	90.03	5.68	85.91	4.19	89.69	4.81
MedNeXt^†^	92.41	4.98	87.75	4.67	83.96	4.51	88.04	4.72
UNETR^†^	92.19	6.17	86.39	5.29	84.48	5.03	87.68	5.49
SwinUNETR^†^	92.71	5.22	87.79	4.42	84.21	4.42	88.24	4.70
SwinUNETR-V2^†^	93.35	5.01	89.65	4.41	85.17	4.41	89.39	4.51
nnU-Net	93.45	4.34	91.93	3.30	87.59	**3.74**	90.99	3.79
UMamba	93.18	4.07	91.78	3.19	87.89	4.01	90.95	3.76
SegMamba	93.30	4.12	**92.49**	3.14	87.21	3.91	91.00	3.72
**Proposed**	**93.56**	**4.06**	92.45	**3.06**	**88.58**	3.83	**91.53**	**3.65**

^†^Results reported in SegMamba (Xing et al., 2024). The bold values indicate the best performance.

Besides, the performance of the proposed model is further compared with several other models that were employed as baseline models in a recent publication on the BraTS dataset (Xing et al., 2024), including SegresNet (Myronenko, 2019), UX-Net (Lee et al., 2023), MedNeXt (Roy et al., 2023), UNETR (Hatamizadeh et al., 2022), SwinUNETR (Hatamizadeh et al., 2021), and SwinUNETR-V2 (He et al., 2023). Note that, for these methods, no re-implementation is performed in the present study due to the lack of open-source code. Consequently, the results reported in Xing et al. (2024) are used in the present study. Besides, only Dice and HD95 are reported in Xing et al. (2024). These two metrics are therefore considered as metrics of the BraTS dataset for all the methods listed in Table 4. It is clear that the performance of our method is superior to those baseline models.

### 3.2 Segmentation results on SWS dataset

**Figure 6:**
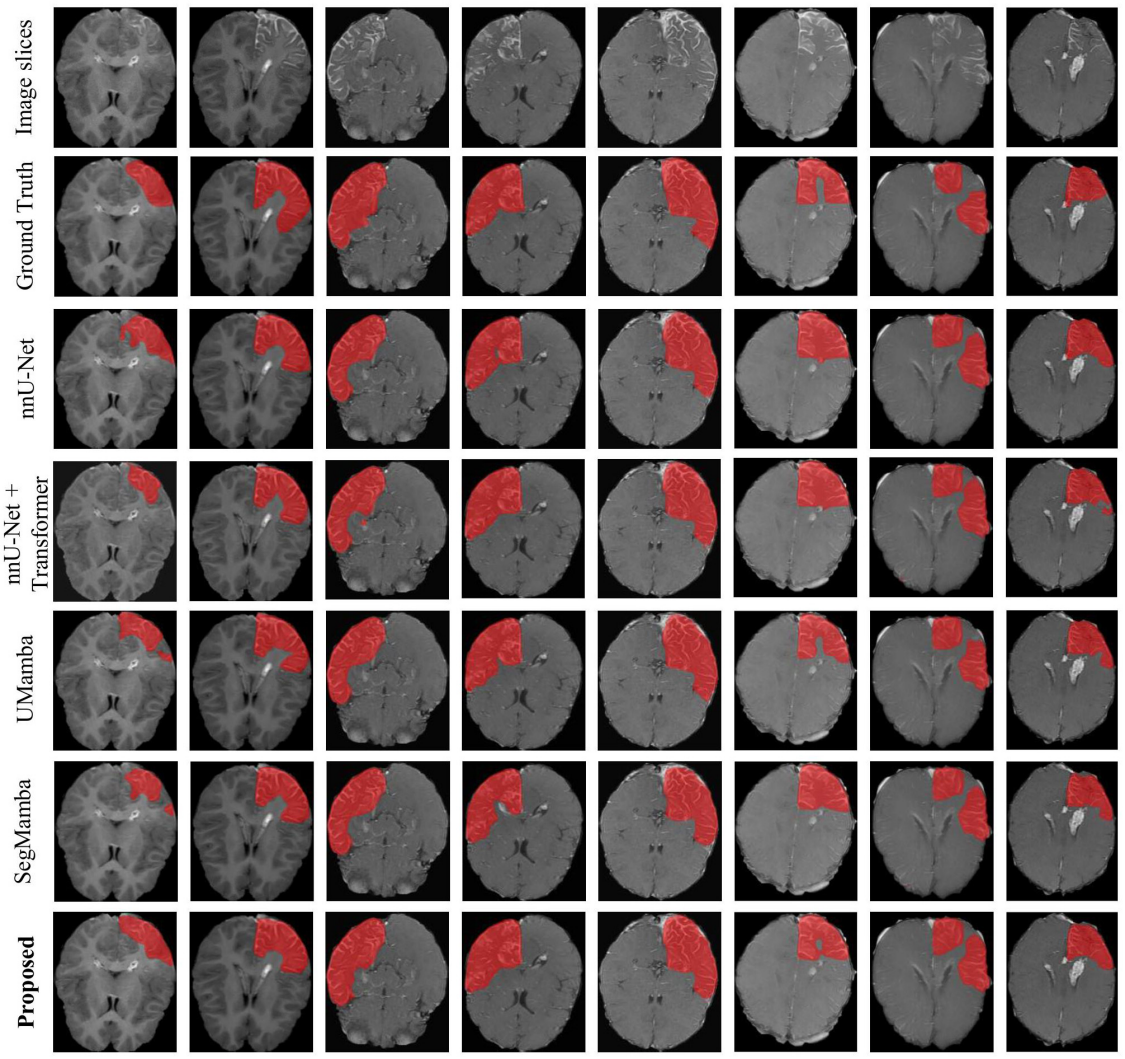
Example of the segmentation results on the SWS dataset.

Figure 6 shows an representation example of the segmentation results on the SWS dataset, produced by the proposed methods as well as the comparison methods including nnU-Net, UMamba, and SegMamba (re-implemented in the present study). It can be observed that the proposed method produces more accurate and coherent LA segmentations, particularly in regions with irregular boundaries.

**Figure 7:**
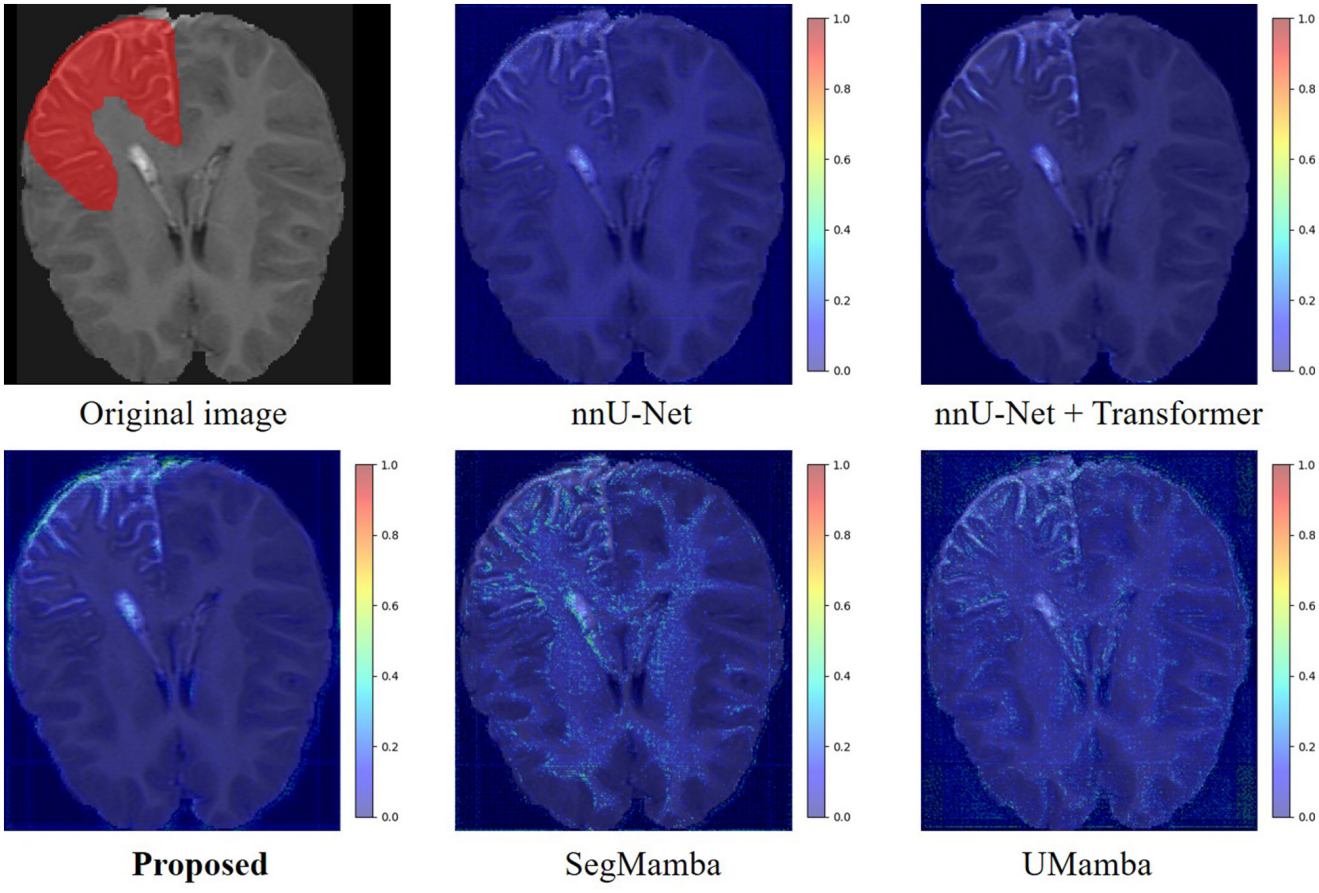
Feature activation maps.

**Figure 8:**
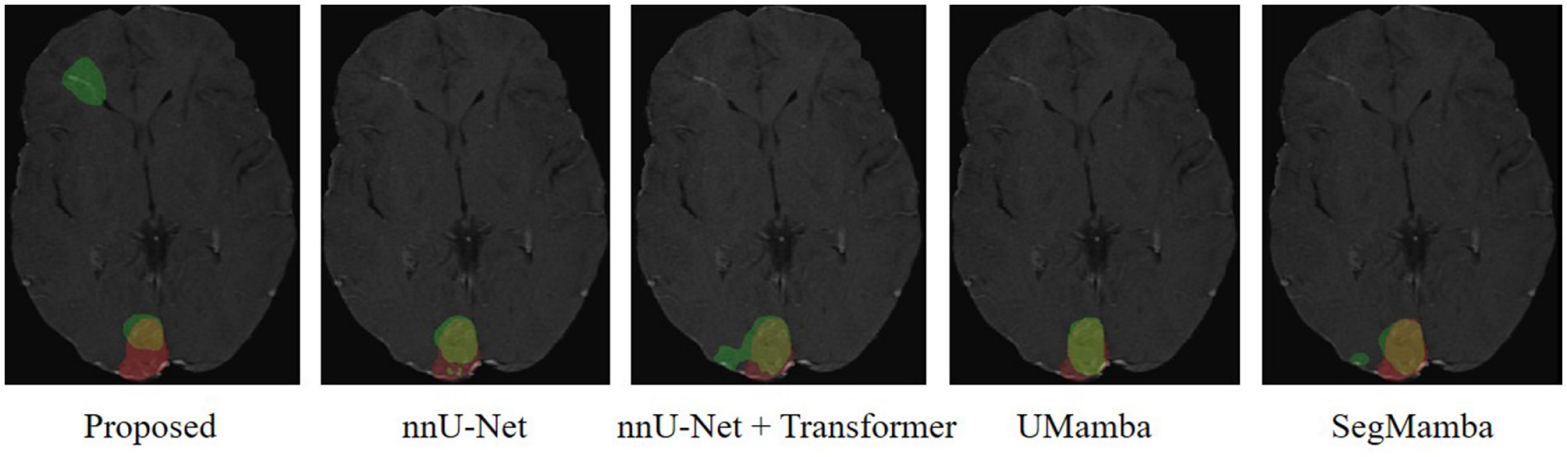
Worst case scenario. Red, Ground Truth; Green, Prediction.

We have employed Grad-CAM (Selvaraju et al., 2017) to visualize the feature activation maps. As shown in the Figure 7, the proposed method exhibits the most distinct and concentrated response in the lesion regions, indicating strong capability for LA localization. In contrast, the nnU-Net and nnU-Net+Transformer models show relatively diffuse activations, while SegMamba and UMamba fail to accurately highlight the lesion areas. However, our model's stronger capability for LA localization may also lead to increased false positive rate in the worst case shown in Figure 8, where the boundary between two different brain areas has similar characteristic as LA, i.e., highlighted (bright) pixels, and is therefore identified as LA area.

The quantitative metrics of the 5-fold cross validation are shown in Table 5. The proposed method achieves the highest mean Dice score of 78.67%, outperforming nnUNet, UMamba, and SegMamba by 1.87%, 2.24%, and 1.62%, respectively. Additionally, the proposed method yields the highest scores in all the other metrics except for specificity and HD95: i.e., sensitivity (78.57%), balanced accuracy (88.87%), accuracy (98.34%), Jaccard index (66.59%), Kappa coefficient (77.95%), volume similarity (92.14%) and Matthews correlation coefficient (78.38%). These results demonstrate the effectiveness of the proposed method in segmenting LA in the MRI images of SWS patients. The slightly lower specificity (99.16%) and HD95 (15.49) may be explained by the higher false positive rate of our model in the worst case scenario, as shown in Figure 8.

**Table 5:** Segmentation results on the SWS dataset.

**Metric**	**nnU-Net**	nnU-Net+T^*^	**UMamba**	**SegMamba**	**Proposed**	**Reader-1**	**Reader-2**
Sen (%)	77.31 ± 14.58	77.23 ± 14.98	77.85 ± 15.33	74.79 ± 17.70	**78.57** **±14.80**	82.38 ± 14.25	79.42 ± 14.12
Spe (%)	99.18 ± 0.61	99.19 ± 0.52	98.91 ± 1.09	**99.29** **±0.58**	99.16 ± 0.65	98.90 ± 0.98	99.12 ± 1.33
BAcc (%)	88.24 ± 7.23	88.21 ± 7.43	88.38 ± 7.51	87.04 ± 8.78	**88.87** **±7.30**	90.64 ± 7.26	89.27 ± 6.91
Acc (%)	98.21 ± 1.02	98.26 ± 0.96	98.05 ± 1.23	98.27 ± 1.03	**98.34** **±0.95**	98.70 ± 0.99	98.85 ± 1.36
JI (%)	64.01 ± 15.59	64.32 ± 16.73	63.55 ± 15.57	64.77 ± 16.65	**66.59** **±14.81**	48.66 ± 18.77	51.41 ± 18.10
Kappa (%)	75.87 ± 13.73	75.92 ± 14.87	75.41 ± 14.07	76.14 ± 16.21	**77.95** **±13.12**	62.41 ± 19.72	65.28 ± 17.96
VS (%)	89.73 ± 9.48	91.79 ± 9.37	89.56 ± 9.47	90.31 ± 8.33	**92.14** **±7.86**	73.63 ± 22.29	79.31 ± 20.98
MCC (%)	76.53 ± 13.19	76.43 ± 14.41	76.10 ± 13.62	76.68 ± 16.00	**78.38** **±12.81**	65.06 ± 17.46	67.54 ± 15.03
HD95 (mm)	17.16 ± 35.93	17.27 ± 38.26	16.83 ± 36.84	**12.83** **±32.16**	15.49 ± 35.39	27.81 ± 41.41	19.87 ± 25.38
Dice (%)	76.80 ± 13.74	76.83 ± 13.83	76.43 ± 14.03	77.05 ± 16.23	**78.67** **±13.11**	62.94 ± 19.98	65.76 ± 18.02

^*^nnU-Net+Transformer (Transformer is only used in the last layer of the encoder). The bold values indicate the best performance.

Note that all the models yield very high specificity and accuracy. A high specificity indicates good ability of the models to detect negative samples (non-LA pixels). Besides, given the relatively low sensitivity (below 80%), the observed high accuracy reveals imbalanced distribution of the two classes of samples (LA and non-LA pixels). This is indeed the case of the present study. As shown in Figure 6, non-LA areas in the MRI images are much larger than LA areas.

To further evaluate the performance of the proposed method for LA detection, two readers (neurologists) were asked to annotate the LA independently, and their results were compared with that of our model. As shown in Table 5, the proposed deep learning model outperforms the two readers in many evaluation metrics, except for sensitivity and accuracy where the readers achieve slightly higher values. These results suggest that while the readers may detect more true positive regions, our method provides more accurate and consistent segmentation results overall.

### 3.3 Ablation study

Ablation study was performed on the SWS dataset in the present study in order to demonstrate the effectiveness of the proposed MSMS architecture as well as the pre-training strategy. The results are reported in Table 6. All these models are embedded in the encoder-decoder architecture shown in Figure 2. Considering only the original Mamba, a Dice score of 76.01% is observed for LA segmentation on the SWS dataset. By adding the multi-scaling (down-sampling) module to the original Mamba, a Dice score of 76.01% (with improvements of 0.89%) is achieved. A combination of the original Mamba and the multi-scanning module yields a Dice score of 75.90% (0.78% improvements compared to the original Mamba).

**Table 6:** Results of ablation study on the SWS dataset.

**Original Mamba**	**Multi-scale**	**Multi-scan**	**Pre-training**	**Dice (%)**
✓				75.12
✓	✓			76.01
✓		✓		75.90
✓			✓	76.43
✓	✓	✓		77.18
✓	✓	✓	✓	**78.67**

The bold values indicate the best performance.

Furthermore, the original Mamba with pre-training on the BraTS dataset produces a Dice score of 76.43% on the SWS dataset, improving 1.31% compared to original Mamba only. Without pre-training, the combination of multi-scale and multi-scan strategies yields a Dice score of 77.18%. Finally, as reported in Table 6, the proposed method integrating multi-scale, multi-scan, and pre-training achieves the best performance, i.e, with a Dice score of 78.67%. These results demonstrate the effectiveness of the MSMS architecture and the pre-training strategy.

### 3.4 Computational complexity

The computational complexity of the proposed method as well as the comparison methods are reported in Table 7. The training and evaluation processes were executed on a high-performance computing node featuring NVIDIA GeForce RTX 3090 graphics processor with CUDA 11.8 acceleration. Due to the addition of Mamba module in each encoding layer, the number of parameters of the proposed method is higher than that of nnU-Net and nnU-Net+Transformer, resulting in slightly higher inference time (0.169 s). However, our model has the lowest Floating Point Operations (892.44 Giga). Notably, for nnU-Net+Transformer, our preliminary results indicate that incorporating a Transformer module at every encoder layer leads to out-of-memory issue. As a consequency, Transformer is only used in the last layer of the encoder, e.g., at the bottleneck.

**Table 7:** Comparison of model's computational complexity.

**Model**	**Inference time^†^ (s/subject)**	**Floating Point Operations (Giga)**	**Parameters (Million)**
nnU-Net	0.055 ± 0.001	928.51	**361.04**
nnU-Net + Transformer^*^	**0.040** **±0.023**	928.95	376.61
UMamba	0.120 ± 0.001	1210.00	728.69
SegMamba	0.172 ± 0.000	965.34	594.40
**Proposed**	0.169 ± 0.000	**892.44**	570.28

^^†^^Mean ± stanard deviation over 10 repeated tests;^^*^^Transformer is only used in the last layer of the encoder. The bold values indicate the best performance.

## 4 Discussion

The aim of the present study is to develop an artificial intelligence (AI) model for automatic identification of LA in the MRI images and thus for automated SWS diagnosis. Such model is of great clinical importance as, currently, the detection of LA in MRI for SWS diagnosis relies on manual inspection by neuroradiologists, which is not only time-consuming and experience-demanding but also lead to low inter-rater agreement. To this end, the encoder-decoder architecture particularly nnU-Net is employed as the basic framework of our model due to its excellent performance in many segmentation tasks (Isensee et al., 2021).

Different from traditional nnU-Net using CNNs in each encoding and decoding layer, Mamba is embedded in each encoding layer in the present study in order to capture long-range dependencies. Based on a selective SSM, Mamba has lower computational complexity as compared to conventional sequential modeling approaches such as RNN and Transformer (Gu and Dao, 2024). Besides, a novel MSMS strategy is proposed to convert the 3-D volumes into sequences as input of the SSM. The MSMS strategy integrates multi-resolution feature fusion with multi-scanning order, enabling robust global context modeling while maintaining linear complexity by explicitly decoupling spatial dependencies across reduced scales and different directions. This lies in the main novelty of the present study.

The ablation results shown in Table 6 demonstrate the effectiveness of the MSMS strategy. Besides, the entire model proposed in the present study outperforms several SOTA models, including nnUNet, UMamba, and SegMamba, in both brain tumor segmentation on the BraTS dataset and LA segmentation on the SWS dataset. Our framework significantly enhances Mamba's feature extraction capability for 3D medical images through multi-scale and multi-scan hierarchical integration, which systematically aggregates discriminative features across varying receptive fields. However, note that the reproduced results for SegMamba on the BraTS dataset (Table 4) are slightly lower than that originally reported in Xing et al. (2024), which may be due to possible difference in the implementation hardware between the present study and Xing et al. (2024).

Furthermore, a main challenge for the application of deep learning to medical data is limited subject size, particularly for rare diseases such as SWS. To overcome this challenge, we introduce a transfer learning approach by pre-training the proposed model on the BraTS dataset and fine-tuning it on the SWS dataset. The ablation results shown in Table 6 illustrate the effectiveness of the pre-training strategy. However, note that the characteristics of the brain tumor (gliomas) in the pre-training dataset (BraTS) differ significantly from that of the LA in the SWS dataset. It is therefore reasonable to expect improved results after pre-training on a dataset similar to SWS. On the other hand, our results may also suggest that pre-training on the BraTS dataset may benefit lesion segmentation in other rare brain diseases with limited dataset but similar imaging madality.

Interesting also to note that the MRI images of the 40 SWS patients are collected with different equipments over a time span of 16 years, which can be ascribed to the rareness of the disease. Nevertheless, with such diverges in the recording equipments and time span, the proposed method produces remarkable segmentation results for the LA, suggesting good generalization ability of the proposed method. In fact, the harmonization technique (Tan et al., 2023) implemented in the pre-processing may contribute significantly to such good generalization ability of the proposed method.

Our results show that the proposed deep learning method outperforms two neurologist in a number of metrics particularly Dice core, which has been widely employed as the primary metric in segmentation tasks. These results suggest that deep learning method may be an alternative to neurologist for the identification of LA and thus the diagnosis of SWS. In fact, The proposed method can either be integrated in a MRI machine or work off-line on a personal computer, indicating the feasibility of auto-segmentation of LA for SWS diagnosis in clinical practice.

Interesting to note that while successfully locating LAs, our model produces higher false positive rate, leading to over-diagnosis in clinical settings. In general, false positives may be more preferable than false negatives in clinical practice, as false negatives yield missed diagnosis but false positives can be excluded by post screening of neuroradiologists. Although neuroradiologists is involved for post screening in case of false positives, the working load is significantly reduced by using our model as a pre-screening tool.

Note also that fully supervised learning is employed in the present study in order to train the coefficients of the deep learning network. The performance of a supervised deep learning model relies strongly on the ground truth. In the present study, annotating the abnormal intracranial vessels in the MRI image is quite difficult and therefore not considered. Instead, the brain area containing the LA is annotated. Besides, the annotation is performed by only one neuroradiologist, limiting the accuracy of the ground truth and therefore the performance of the proposed model for LA segmentation. On the one hand, this may partially explain the observed lowered segmentation results on the SWS dataset as compared with that on the BraTS dataset. On the other hand, self-supervised learning (Krishnan et al., 2022) or few-shot learning (Song et al., 2023) with less dependence on the ground truth annotation may be an interesting direction for future studies on LA segmentation for SWS diagnosis.

## 5 Conclusion

In the present study, we propose a MSMS-based Mamba, embedded into an encoder-decoder architecture, for the identification of LA in the brain MRI images and thus for automated diagnosis of SWS. A pre-training strategy is employed to overcome the challenges introduced by small subject size. Our results show promising segmentation performance of the proposed model on the public BraTS dataset as well as our 40 SWS dataset, outperforming several SOTA models and two independent readers. These findings suggest the feasibility of using AI tool for automatic identification of LA, providing useful information for clinical SWS diagnosis that is currently performed by time-consuming manual inspection. Future studies may focus on self-supervised learning and/or few-shot learning that depend less on the ground truth in order to reduce the requirement of accurate annotations.

## Data Availability

The raw data supporting the conclusions of this article will be made available by the authors under reasonable request.

## References

[B1] Bar C. Pedespan J.-M. Boccara O. Garcelon N. Levy R. Grévent D. . (2020). Early magnetic resonance imaging to detect presymptomatic leptomeningeal angioma in children with suspected sturge-weber syndrome. Dev. Med. Child Neurol. 62, 227–233. doi: 10.1111/dmcn.14253 31050360

[B2] Chen W. Tan X. Zhang J. Du G. Fu Q. Jiang H. (2025). Mlg: A mixed local and global model for brain tumor classification. Front. Neurosci. 19:1618514. doi: 10.3389/fnins.2025.1618514 40678755 PMC12267167

[B3] Chicco D. Jurman G. (2020). The advantages of the matthews correlation coefficient (MCC) over F1 score and accuracy in binary classification evaluation. BMC Genomics 21:6. doi: 10.1186/s12864-019-6413-7 31898477 PMC6941312

[B4] Chmura Kraemer H. Periyakoil V. S. Noda A. (2002). Kappa coefficients in medical research. Stat. Med. 21, 2109–2129. doi: 10.1002/sim.1180 12111890

[B5] Çiçek Ö. Abdulkadir A. Lienkamp S. S. Brox T. Ronneberger O. (2016). “3D U-Net: learning dense volumetric segmentation from sparse annotation,” in Medical Image Computing and Computer-Assisted Intervention (Athens: Springer), 424–432. doi: 10.1007/978-3-319-46723-8_49

[B6] Comi A. M. (2007). Update on sturge-weber syndrome: diagnosis, treatment, quantitative measures, and controversies. Lymphat. Res. Biol. 5, 257–264. doi: 10.1089/lrb.2007.1016 18370916

[B7] Dosovitskiy A. Beyer L. Kolesnikov A. Weissenborn D. Zhai X. Unterthiner T. . (2021). “An image is worth 16x16 words: transformers for image recognition at scale,” in International Conference on Learning Representations, 611–631.

[B8] Gu A. Dao T. (2024). “Mamba: linear-time sequence modeling with selective state spaces,” in First Conference on Language Modeling (Philadelphia, PA), 1–32.

[B9] Gu A. Goel K. Re C. (2022). “Efficiently modeling long sequences with structured state spaces,” in International Conference on Learning Representations, 14323–14343.

[B10] Hatamizadeh A. Nath V. Tang Y. Yang D. Roth H. R. Xu D. (2021). “Swin unetr: Swin transformers for semantic segmentation of brain tumors in MRI images,” in International MICCAI Brainlesion Workshop (Springer), 272–284. doi: 10.1007/978-3-031-08999-2_22

[B11] Hatamizadeh A. Tang Y. Nath V. Yang D. Myronenko A. Landman B. . (2022). “Unetr: transformers for 3d medical image segmentation,” in Proceedings of the IEEE/CVF Winter Conference on Applications of Computer Vision (Waikoloa, HI: IEEE), 574–584. doi: 10.1109/WACV51458.2022.00181

[B12] He Y. Nath V. Yang D. Tang Y. Myronenko A. Xu D. (2023). “Swinunetr-v2: Stronger swin transformers with stagewise convolutions for 3d medical image segmentation,” in International Conference on Medical Image Computing and Computer-Assisted Intervention (Vancouver, BC: Springer), 416–426. doi: 10.1007/978-3-031-43901-8_40

[B13] Huttenlocher D. P. Klanderman G. A. Rucklidge W. J. (1993). Comparing images using the Hausdorff distance. IEEE Trans. Pattern Anal. Mach. Intell. 15, 850–863. doi: 10.1109/34.232073

[B14] Isensee F. Jaeger P. F. Kohl S. A. Petersen J. Maier-Hein K. H. (2021). nnU-Net: a self-configuring method for deep learning-based biomedical image segmentation. Nat. Methods 18, 203–211. doi: 10.1038/s41592-020-01008-z 33288961

[B15] Kazerooni A. F. Khalili N. Liu X. Haldar D. Jiang Z. Anwar S. M. . (2024). The brain tumor segmentation (brats) challenge 2023: focus on pediatrics (cbtn-connect-dipgr-asnr-miccai brats-peds). ArXiv:2305.17033. doi: 10.48550/arXiv.2305.1703337292481

[B16] Krishnan R. Rajpurkar P. Topol E. J. (2022). Self-supervised learning in medicine and healthcare. Nat. Biomed. Eng. 6, 1346–1352. doi: 10.1038/s41551-022-00914-1 35953649

[B17] Lee C.-Y. Xie S. Gallagher P. Zhang Z. Tu Z. (2015). “Deeply-supervised nets,” in Artificial Intelligence and Statistics (Lille: Pmlr), 562–570.

[B18] Lee H. H. Bao S. Huo Y. Landman B. A. (2023). “3D UX-Net: a large kernel volumetric convnet modernizing hierarchical transformer for medical image segmentation,” in International Conference on Learning Representations (Kigali), 21891–21905. PMC13597054 42775365

[B19] Liu J. Yang H. Zhou H.-Y. Xi Y. Yu L. Li C. . (2024). “Swin-umamba: Mamba-based unet with imagenet-based pretraining,” in International Conference on Medical Image Computing and Computer-Assisted Intervention (Marrakesh: Springer), 615–625. doi: 10.1007/978-3-031-72114-4_59

[B20] Liu Y. Tian Y. Zhao Y. Yu H. Xie L. Wang Y. . (2024). Vmamba: visual state space model. Adv. Neural Inform. Process. Syst. 37, 103031–103063. doi: 10.48550/arXiv.2401.10166

[B21] Ma J. Li F. Wang B. (2024). U-Mamba: enhancing long-range dependency for biomedical image segmentation. arXiv:2401.04722. doi: 10.48550/arXiv.2401.04722

[B22] Myronenko A. (2019). “3D MRI brain tumor segmentation using autoencoder regularization,” in Brainlesion: Glioma, Multiple Sclerosis, Stroke and Traumatic Brain Injuries: 4th International Workshop, BrainLes 2018, Held in Conjunction with MICCAI 2018, Granada, Spain, September 16, 2018, Revised Selected Papers, Part II 4 (Granada: Springer), 311–320. doi: 10.1007/978-3-030-11726-9_28

[B23] Raghu M. Unterthiner T. Kornblith S. Zhang C. Dosovitskiy A. (2021). Do vision transformers see like convolutional neural networks? Adv. Neural Inform. Process. Syst. 34, 12116–12128. doi: 10.48550/arXiv.2108.08810

[B24] Ramirez E. L. Jülich K. (2024). “Sturge-weber syndrome: an overview of history, genetics, clinical manifestations, and management,” in Seminars in Pediatric Neurology, Volume 51 (Philadelphia, PA: Elsevier), 101151. doi: 10.1016/j.spen.2024.101151 39389653

[B25] Ronneberger O. Fischer P. Brox T. (2015). “U-Net: convolutional networks for biomedical image segmentation,” in Medical Image Computing and Computer-Assisted Intervention (Munich: Springer), 234–241. doi: 10.1007/978-3-319-24574-4_28

[B26] Roy S. Koehler G. Ulrich C. Baumgartner M. Petersen J. Isensee F. . (2023). “Mednext: transformer-driven scaling of convnets for medical image segmentation,” in International Conference on Medical Image Computing and Computer-Assisted Intervention (Vancouver, BC: Springer), 405–415. doi: 10.1007/978-3-031-43901-8_39

[B27] Selvaraju R. R. Cogswell M. Das A. Vedantam R. Parikh D. Batra D. (2017). “Grad-cam: visual explanations from deep networks via gradient-based localization,” in Proceedings of the IEEE International Conference on Computer Vision (Venice: IEEE), 618–626. doi: 10.1109/ICCV.2017.74

[B28] Shi Y. Dong M. Xu C. (2024). Multi-scale vmamba: hierarchy in hierarchy visual state space model. Adv. Neural Inform. Process. Syst. 37, 25687–25708. doi: 10.48550/arXiv.2405.14174

[B29] Soh W. K. Rajapakse J. C. (2023). Hybrid unet transformer architecture for ischemic stoke segmentation with mri and ct datasets. Front. Neurosci. 17:1298514. doi: 10.3389/fnins.2023.1298514 38105927 PMC10723803

[B30] Song Y. Wang T. Cai P. Mondal S. K. Sahoo J. P. (2023). A comprehensive survey of few-shot learning: evolution, applications, challenges, and opportunities. ACM Comput. Surv. 55, 1–40. doi: 10.1145/3582688

[B31] Sudarsanam A. Ardern-Holmes S. L. (2014). Sturge-weber syndrome: from the past to the present. Eur. J. Paediatr. Neurol. 18, 257–266. doi: 10.1016/j.ejpn.2013.10.003 24275166

[B32] Sujansky E. Conradi S. (1995). Outcome of sturge-weber syndrome in 52 adults. Am. J. Med. Genet. 57, 35–45. doi: 10.1002/ajmg.1320570110 7645596

[B33] Tan T. Tegzes P. Török L. I. Ferenczi L. Avinash G. B. Ruskó L. . (2023). Image Harmonization for Deep Learning Model Optimization. Technical Report. US Patent 11669945.

[B34] Thomas-Sohl K. A. Vaslow D. F. Maria B. L. (2004). Sturge-weber syndrome: a review. Pediatr. Neurol. 30, 303–310. doi: 10.1016/j.pediatrneurol.2003.12.015 15165630

[B35] Ulyanov D. Vedaldi A. Lempitsky V. (2016). Instance normalization: the missing ingredient for fast stylization. arXiv:1607.08022. doi: 10.48550/arXiv.1607.08022

[B36] Xing Z. Ye T. Yang Y. Liu G. Zhu L. (2024). “Segmamba: long-range sequential modeling mamba for 3d medical image segmentation,” in International Conference on Medical Image Computing and Computer-Assisted Intervention (Marrakesh: Springer), 578–588. doi: 10.1007/978-3-031-72111-3_54

[B37] Xu J. Li Z. Du B. Zhang M. Liu J. (2020). “Reluplex made more practical: leaky ReLU,” in 2020 IEEE Symposium on Computers and communications (ISCC) (Rennes: IEEE), 1–7. doi: 10.1109/ISCC50000.2020.9219587

[B38] Yushkevich P. A. Piven J. Hazlett H. C. Smith R. G. Ho S. Gee J. C. . (2006). User-guided 3D active contour segmentation of anatomical structures: significantly improved efficiency and reliability. Neuroimage 31, 1116–1128. doi: 10.1016/j.neuroimage.2006.01.015 16545965

[B39] Zhang T. Zhang X. Shi J. Wei S. (2019). Depthwise separable convolution neural network for high-speed sar ship detection. Remote Sens. 11:2483. doi: 10.3390/rs11212483

[B40] Zhu Z. Wang Z. Qi G. Zhao Y. Liu Y. (2025). Visually stabilized mamba U-shaped network with strong inductive bias for 3-D brain tumor segmentation. IEEE Trans. Instrument. Measur. 74, 1–11. doi: 10.1109/TIM.2025.3551581

